# **Risk factors for intraoperative hypothermia during laparoscopic surgery**: **A systematic review and meta-analysis**

**DOI:** 10.1371/journal.pone.0328282

**Published:** 2025-07-17

**Authors:** Hao Wang, Alei Wang, Xiaoyan Song, Jianying Luo, Peihong Zhang

**Affiliations:** Sixth People’s Hospital South Campus Affiliated to Shanghai Jiaotong University, Shanghai, China; IRCCS: IRCCS Ospedale San Raffaele, ITALY

## Abstract

**Objectives:**

To systematically evaluate the risk factors for intraoperative hypothermia in patients undergoing laparoscopic surgery globally; and to provide information on how to prevent complications and, should they occur, how to intervene.

**Methods:**

This study was registered in the International Prospective Register of Systematic Reviews (PROSPERO; No. CRD42024555506). We searched the following databases: PubMed, Cumulative Index of Nursing and Allied Health Literature (CINAHL), Web of Science Core Collection, EMBASE, China National Knowledge Infrastructure (CNKI), and Wanfang. Data on risk factors for hypothermia during laparoscopic surgery were systematically collected through June 1, 2024. After evaluating references that met Newcastle–Ottawa scale (NOS) or Agency for Healthcare Research and Quality (AHRQ) inclusion criteria, we performed a meta-analysis of the extracted data using RevMan version 5.4.

**Results:**

We included 11 studies with a cumulative sample size of 3550 cases and extracted 14 risk factors. Meta-analysis results showed that age (odds ratio [OR], 1.02; 95% confidence interval [CI], 1.00–1.03; *P* = 0.04), total amount of intraoperative CO_2_ injected into the abdominal cavity > 200 L (OR, 1.5; 95% CI, 1.30–1.81; *P* < 0.001), duration of operation > 120 min (OR, 2.32; 95% CI, 2.03–2.65; *P* < 0.001), duration of anesthesia > 150 min (OR, 1.55; 95% CI, 1.26–1.92; *P* < 0.001), intravenous (IV)–fluid volume>1500 mL (OR = 1.77; 95% CI, 1.48–2.12; *P* < 0.001), and intraoperative blood loss ≥ 150 mL (OR, 1.66; 95% CI, 1.27–2.17; *P* < 0.001) were risk factors for intraoperative hypothermia.

**Conclusions:**

We found that age, total amount of CO_2_ injected into the abdominal cavity during the operation, operation duration, anesthesia duration, IV-fluid volume, and intraoperative blood loss to be risk factors for intraoperative hypothermia in patients undergoing laparoscopic surgery. Given the limitations of the available literature’s quantity and quality, our conclusions should be verified by higher-quality studies.

## Introduction

Given recent advances in surgical technology, the proportion of endoscopic surgeries has gradually increased, meaning that the occurrence of intraoperative hypothermia during laparoscopic surgery has come to require urgent attention [1]. Studies have shown that the number of surgeries for gastrointestinal [2], hepatobiliary [3], urinary [4], and other diseases using laparoscopic technology now far exceeds the number of traditional open procedures. In both laparoscopic and open surgeries, unplanned hypothermia can occur. This generally refers to a patient’s core temperature dropping to < 36°C during anesthesia and surgery, potentially causing chills, abnormal coagulation, delayed recovery from anesthesia, wound infection, and other adverse events. The global incidence of inadvertent perioperative hypothermia (IPH) ranges from 4% to 72% and can climb as high as 90% [5–7], seriously affecting patient prognosis. Although the mechanisms and causes of IPH remain unclear, it is generally believed to be closely related to the patient’s individual constitution, the method and timing of anesthesia, operation time, and other factors [8]. Some studies have suggested that its incidence in laparoscopic surgery is higher than that in traditional open surgery [9,10]. Many scholars have conducted studies on risk factors for hypothermia during laparoscopic surgery and achieved reportable results. However, because sample sources, sample sizes, and the quality of different studies vary, these results tend to be one sided and even conflicting; therefore, they cannot provide effective guidance on IPH. The purpose of our study was to screen all of the relevant high-quality literature and conduct a meta-analysis of the more reliable and extensive studies to provide guidance for nursing staff on the prevention and treatment of IPH.

## Methods

### Search strategy

We searched the following databases: PubMed, Cumulative Index of Nursing and Allied Health Literature (CINAHL), Web of Science Core Collection, Embase, China National Knowledge Infrastructure (CNKI), and Wanfang. Data on risk factors for hypothermia during laparoscopic surgery were systematically collected. The search was conducted using a combination of subject words and free text without setting a time limit. Search strategy was as follows:the theme was (“risk factors” [Title/Abstract] OR “influencing factors” [Title/Abstract] OR “risk” [Title/Abstract] OR “related factors” [Title/Abstract]) AND (“laparoscopy” [Title/Abstract] OR “Surgery” [Title/Abstract] OR “intraoperative” [Title/Abstract]) AND (“hypothermia” [Medical Subject Headings (MeSH)]). The search was completed on June 1, 2024.

### Inclusion and exclusion criteria

Inclusion criteria were as follows: (1) subjects were patients ≥ 18 years of age undergoing laparoscopic surgery; (2) the study included an analysis of risk factors for IPH; (3) study type was case–control, cross-sectional, or cohort, and the study provided odds ratios (ORs) with 95% confidence intervals (CIs); (4) the study’s outcome index reported whether hypothermia (< 36°C) occurred during the operation; and (5) the study was in the Chinese or English language. Exclusion criteria were as follows: (1) case reports, reviews, conference abstracts, and systematic reviews; (2) incomplete or unavailable literature; (3) duplicate publications; (4) low-quality paper with a Newcastle-Ottawa Scale (NOS) score < 5; and (5) low-quality paper with an Agency for Healthcare Research and Quality (AHRQ) score < 4.

### Data extraction

Literature was screened and data were extracted using NoteExpress software (Apconic Software Pvt Ltd, Pune, India) by author, title, and publication year. Two trained researchers who read the title, abstract, and full text independently screened and evaluated the studies, extracted the data, and performed cross-checking. When these two researchers’ opinions conflicted, a third researcher helped resolve the conflict through discussion and/or consultation. In addition, when necessary, the authors of the studies were contacted to obtain further data. Extraction of data was carried out in reference to the table formulated by JBI, which included basic information (author, publication time, study type, study site, sample size, exclusion criteria) and analysis indicators (risk factors, number of cases, assignment criteria, OR value, 95% CI).

### Methodological-quality assessment

We used the NOS to evaluate case–control and cohort studies, ultimately including those with scores of ≥ 5 stars [11]. In the United States, healthcare quality and research institutions (e.g., AHRQ) recommend quality appraisal standards to evaluate cross-sectional studies that will score more than 4 points; we included only such high-quality studies in our meta-analysis [12]. As stated in the previous subsection, two researchers independently evaluated the literature on quality, resolving disagreements through discussion or consultation with a third researcher.

### Statistical analysis

Review Manager v5.4 (Cochrane, London, UK) and Stata v1.4 (StataCorp., College Station, TX, USA) were used for meta-analysis. The *Q* test was used to determine heterogeneity among studies. When *I*^2^ < 50% and *P* > 0.1, there was no heterogeneity, and we used a fixed-utility model for analysis. When *I*^2^ > 50% or *P* < 0.1, we performed sensitivity and subgroup analyses to find and eliminate heterogeneous sources. If heterogeneity still could not be eliminated, a random-effects model was used for analysis. The combined effect was represented by ORs and 95% CIs, and *P* < 0.05 was considered statistically significant. We conducted Egger’s test to assess publication bias within each study. Relatively few studies included such individual influencing factors such as age, intraoperative irrigation volume, and intraoperative blood loss, making publication bias analysis impossible. Therefore, in this study we examined only body mass index (BMI), operation time, and total CO_2_ injected into the abdominal cavity during the operation. Operation time, anesthesia time, intraoperative intravenous (IV)–fluid intake, and preoperative baseline core body temperature were analyzed for publication bias. All Egger’s test results showed no publication bias (*P* > 0.05).

## Results

### Literature selection

**Fig 1:**
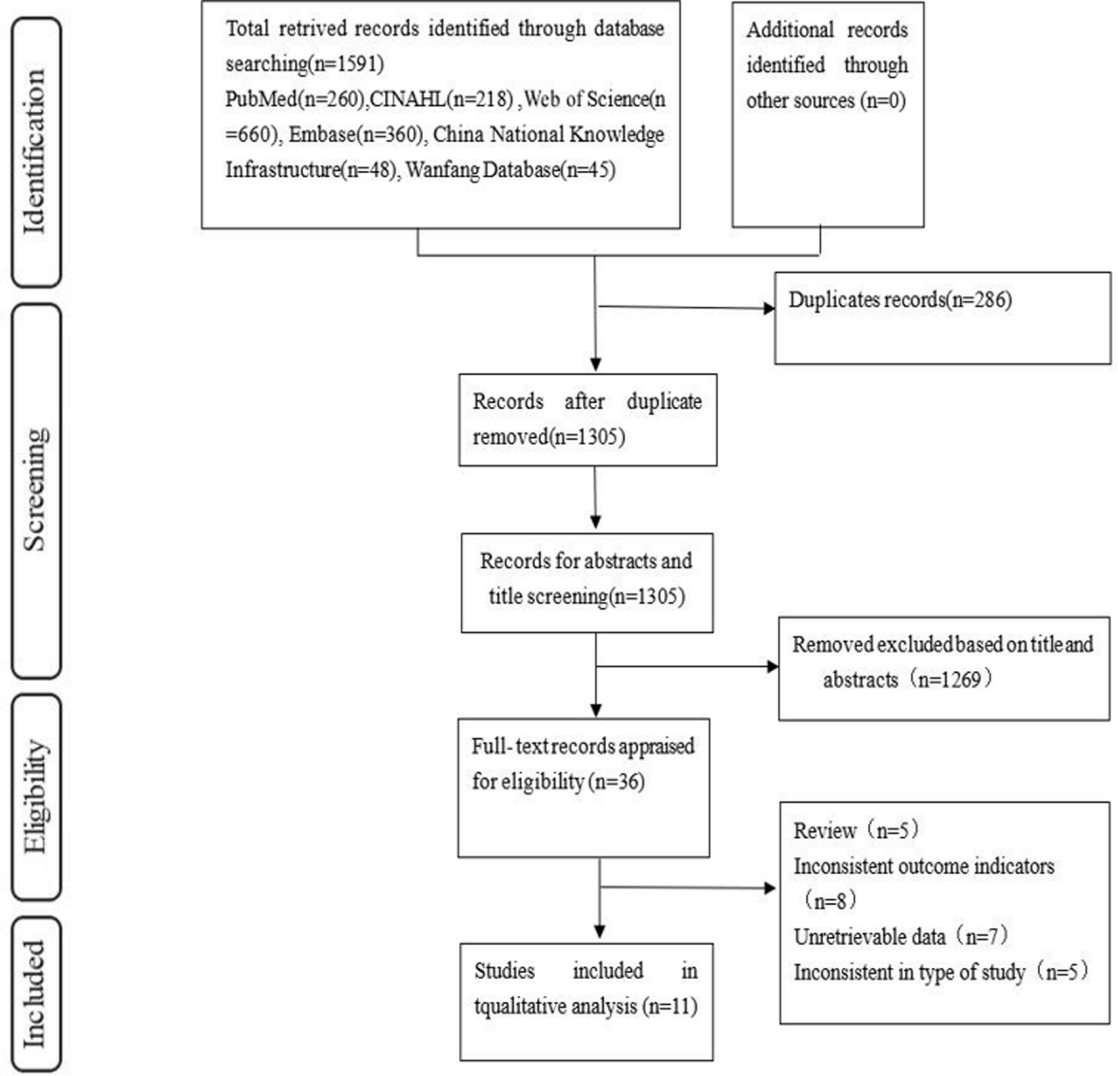
Selection process flowchart.

During the literature retrieval and quality evaluation phase, 1591 relevant studies were retrieved from the database; 286 were retained after removal of duplicate studies using NoteExpress; and 1305 studies with inconsistent reviews, inconsistent research methods and content, unavailable data, and low-quality grades were removed by reading the title, abstract, and full text. Ultimately, we included 11 studies [9–18]. The selection process is shown in Fig 1.

### Study characteristics

The 11 included studies were conducted in two countries, China (n = 10) and Korea (n = 1). Of these, four were case–control studies, three were cross-sectional studies, and four were cohort studies; all had NOS scores of 6–7 or AHRQ scores of 7–8. A total of 3550 subjects were included, and 14 influencing factors were extracted. The basic characteristics and quality evaluations of the studies are shown in Table 1.

**Table 1:** Basic information on included articles.

Author, year	Country	Hypothermia cases/total cases	Type of operation	Core body temperature measurement site	Study design	Influencing factors	Quality evaluation	
							AHRQ score	NOS score
Zhao Z, 2018 [13]	China	104/184	Rectal	Nasopharyngeal	Cross-sectional	4, 6, 8	7	—
Qi F, 2019 [14]	China	35/62	Colorectal	Nasopharyngeal	Case–control	6, 8, 9	—	6
Pu Y, 2019 [15]	China	116/264	Abdominal organ	Nasopharyngeal	Case–control	6, 11, 12, 13	—	7
Ma GL, 2020 [16]	China	49/384	Uterine	Tympanic membrane	Case–control	4, 5, 6, 8, 10	—	7
Zhao D, 2021 [17]	China	68/168	Uterine	Nasopharyngeal	Case–control	2, 4, 5, 6, 8	—	7
Chen HY, 2021 [18]	China	119/383	Uterine	Tympanic membrane	Cross-sectional	5, 7, 8	8	—
Chen HL, 2021 [19]	China	200/690	Abdominal	Nasopharyngeal	Cohort	1, 2, 5, 7, 11	8	—
Sung-Ae C, 2022 [20]	Korea	145/516	Gyneco-logical	Tympanic membrane	Cohort	2, 3, 8, 11, 14	—	7
Liu L, 2022 [21]	China	118/275	Colorectal	Nasopharyngeal	Cohort	4, 6, 8, 10	—	7
Fang M, 2023 [22]	China	51/408	Uterine	Unspecified	Cohort	1, 4, 5, 6, 8, 10	—	6
Shen CY, 2024 [23]	China	114/216	Patients underwent laparosco-pic surgery	Nasopharyngeal	Cross-sectional	2, 5, 11, 13	7	—

Notes: 1, age; 2, body mass index; 3, baseline heart rate; 4, total CO2 injected into the abdominal cavity > 200 L; 5, operation time > 120 min; 6, anesthesia time > 150 min; 7, volume of irrigation; 8, IV fluid administered > 1500 mL; 9, intraoperative blood transfusion volume; 10, intraoperative blood loss ≥ 150 mL; 11, basal body temperature; 12, type of operation; 13, operating room temperature; 14, intraoperative nicardipine.

### Involvement of patients and the public

No patients were involved in this study.

### Results of meta-analysis

#### Age.

**Fig 2:**
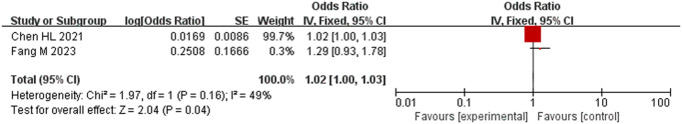
Meta-analysis of the effect of age on hypothermia.

Age was included in two studies [19,22] comprising 1098 subjects, which reported a relationship between age and intraoperative hypothermia. Analysis of the extracted data showed no heterogeneity between these two studies (*P* = 0.16, *I*^2^ = 49%); therefore, the fixed-effects model was used for meta-analysis. The results showed that age was a risk factor for intraoperative hypothermia (OR = 1.02, 95% CI [1.00, 1.03], P = 0.04) (Fig 2).

#### Body mass index.

**Fig 3:**
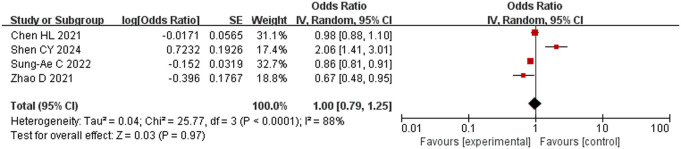
Meta-analysis of the effect of BMI on hypothermia.

Four studies [17,19,20,23] comprising 1590 subjects were included, and they were highly heterogeneous (*P* < 0.001, *I*^2^ = 88%). Sensitivity analysis showed that Sung-Ae *et al.* [20] had a strong effect on the results, but much heterogeneity still remained after that study was removed (*P* = 0.03, *I*^2^ = 70%). Therefore, a random-effects model was used for meta-analysis. The results showed that BMI was not a risk factor for intraoperative hypothermia OR = 1.00, 95% CI [0.79, 1.25], P = 0.07) (Fig 3).

#### Total amount of CO_2_ injected into the abdominal cavity during the operation.

**Fig 4:**
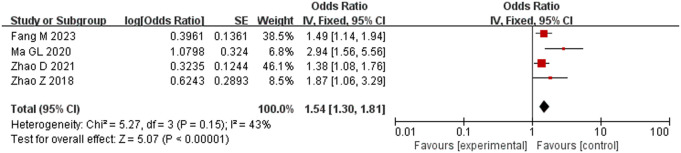
Meta-analysis of the effect of intraoperatively injected CO_2_ on hypothermia.

Five studies [13,16,17,21,22] comprising 1419 subjects were included, and there was considerable heterogeneity among them (*P* = 0.06, *I*^2^ = 56%). Sensitivity analysis determined that Zhao *et al.* [17] had a strong effect on the results; after it was removed, no heterogeneity remained among the other four studies (*P* = 0.15, I^2^ = 43%). Therefore, meta-analysis was conducted using the fixed-effects model. The results showed that the total amount of CO_2_ injected into the abdominal cavity during surgery was an influential factor in the occurrence of hypothermia: the incidence of hypothermia was higher in patients who received a total of CO_2_ > 200 L, and this finding was statistically significant (OR = 1.54, 95% CI [1.30, 1.81], P < 0.001) (Fig 4).

#### Operation time.

**Fig 5:**
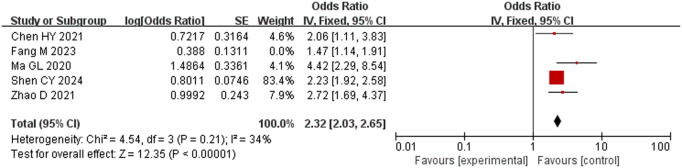
Meta-analysis of the effect of operation time on hypothermia.

Six studies [16–19,22,23] comprising 2249 subjects were included, and there was considerable heterogeneity among them (*P* < 0.001, *I*^2^ = 97%). Sensitivity analysis indicated that Chen *et al.* [18] had a significant effect on the results. After this study was removed, heterogeneity disappeared (*P* = 0.21, *I*^2^ = 34%); therefore, the fixed-effects model was adopted for meta-analysis. The results showed that operation time was a risk factor for hypothermia: patients with operation time > 120 min had a higher risk of hypothermia. This difference was statistically significant (OR = 2.32, 95% CI [2.03, 2.65], P < 0.001) (Fig 5).

#### Anesthesia time.

**Fig 6:**
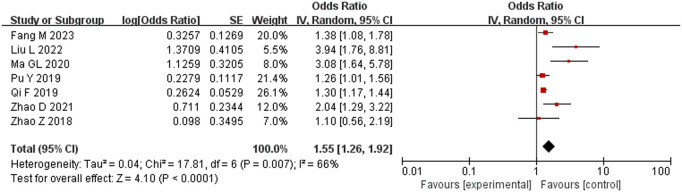
Meta-analysis of the effect of anesthesia time on hypothermia.

A total of 7 studies [13–17,21,22] comprising 1745 subjects were included, and there was considerable heterogeneity among them (*P* = 0.007, *I*^2^ = 66%). Sensitivity analysis determined that the study by Qi et al. [14] had a strong effect on the results, but great heterogeneity still remained after it was removed (*P* = 0.007, *I*^2^ = 54%). Therefore, the random-effects model was used for meta-analysis. The results showed that anesthesia time > 150 min was a high-risk factor for hypothermia, and the difference was statistically significant (OR = 1.55, 95% CI [1.26,1.92], P < 0.001) (Fig 6).

#### Intraoperative intravenous-fluid intake.

**Fig 7:**
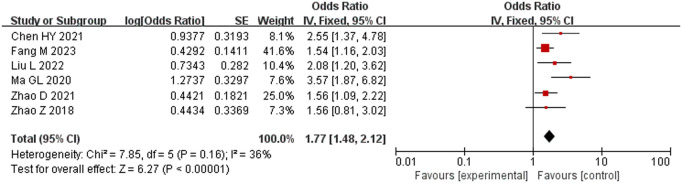
Meta-analysis of the effect of intraoperative intravenous-fluid replenishment on hypothermia.

Eight studies [13,14,16–18,20–22] comprising 2380 subjects were included, and there was considerable heterogeneity among them (*P* < 0.001, *I*^2^ = 86%). Sensitivity analysis found that Sung [20] and Qi [14] had stronger effects on the results; after these two studies were removed, no heterogeneity remained (*P* = 0.16, *I*^2^ = 36%). The fixed-effects model was selected for meta-analysis by gender. The results showed that intraoperative infusion volume was an influential factor in the occurrence of hypothermia: patients with infusion volumes > 1500 mL had a statistically significantly higher risk of hypothermia (OR = 1.77, 95% CI [1.48, 2.12], P < 0.001) (Fig 7).

#### Intraoperative irrigation volume.

**Fig 8:**
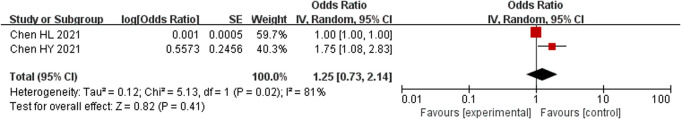
Meta-analysis of the effect of intraoperative irrigation volume on hypothermia.

Two studies comprising 1073 subjects were included [18,19]. Because there was considerable heterogeneity between them (*P* = 0.02, *I*^2^ = 81%), the random-effects model was used for meta-analysis. The results showed that intraoperative irrigation volume was not a risk factor for intraoperative hypothermia (OR = 1.25, 95% CI [0.73, 2.14], P = 0.41) (Fig 8).

#### Intraoperative blood loss.

**Fig 9:**
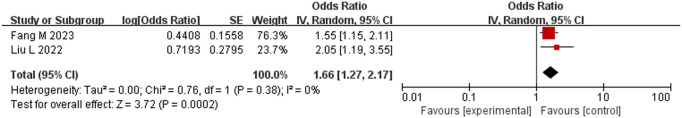
Meta-analysis of the effect of intraoperative blood loss on hypothermia.

Three studies [16,21,22] comprising 1067 subjects were included, and there was considerable heterogeneity among them (*P* = 0.001, I^2^ = 85%). Sensitivity analysis found that Liu *et al.* [21] had a significant effect on the results. Removal of this study also removed the heterogeneity (*P* = 0.38, *I*^2^ = 0%); therefore, the fixed-effects model was adopted for meta-analysis. The results showed that intraoperative blood loss ≥ 150 mL was a risk factor for intraoperative hypothermia (OR = 1.66, 95% CI [1.27, 2.17], P < 0.001) (Fig 9).

#### Preoperative baseline core body temperature.

**Fig 10:**
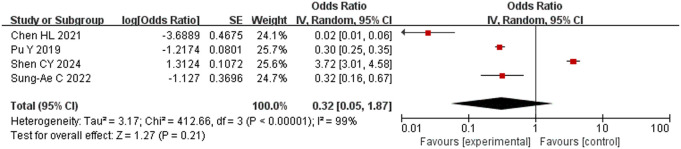
Meta-analysis of the effect of preoperative baseline core body temperature on hypothermia.

Four studies [15,19,20,23] comprising 1686 subjects were included. All reported a relationship between preoperative baseline core body temperature and intraoperative hypothermia. Data extracted for analysis showed considerable heterogeneity among these studies (*P* < 0.001, *I*^2^ = 99%). Pu *et al.* [15] was found to have a significant effect on the results, but much heterogeneity still remained after its removal (*P* < 0.001, *I*^2^ = 93%). Therefore, the random-effects model was used for meta-analysis. The results showed that preoperative baseline core body temperature was not a risk factor for intraoperative hypothermia (OR = 0.32, 95% CI [0.05, 1.87], P = 0.21) (Fig 10).

#### Publication bias.

**Fig 11:**
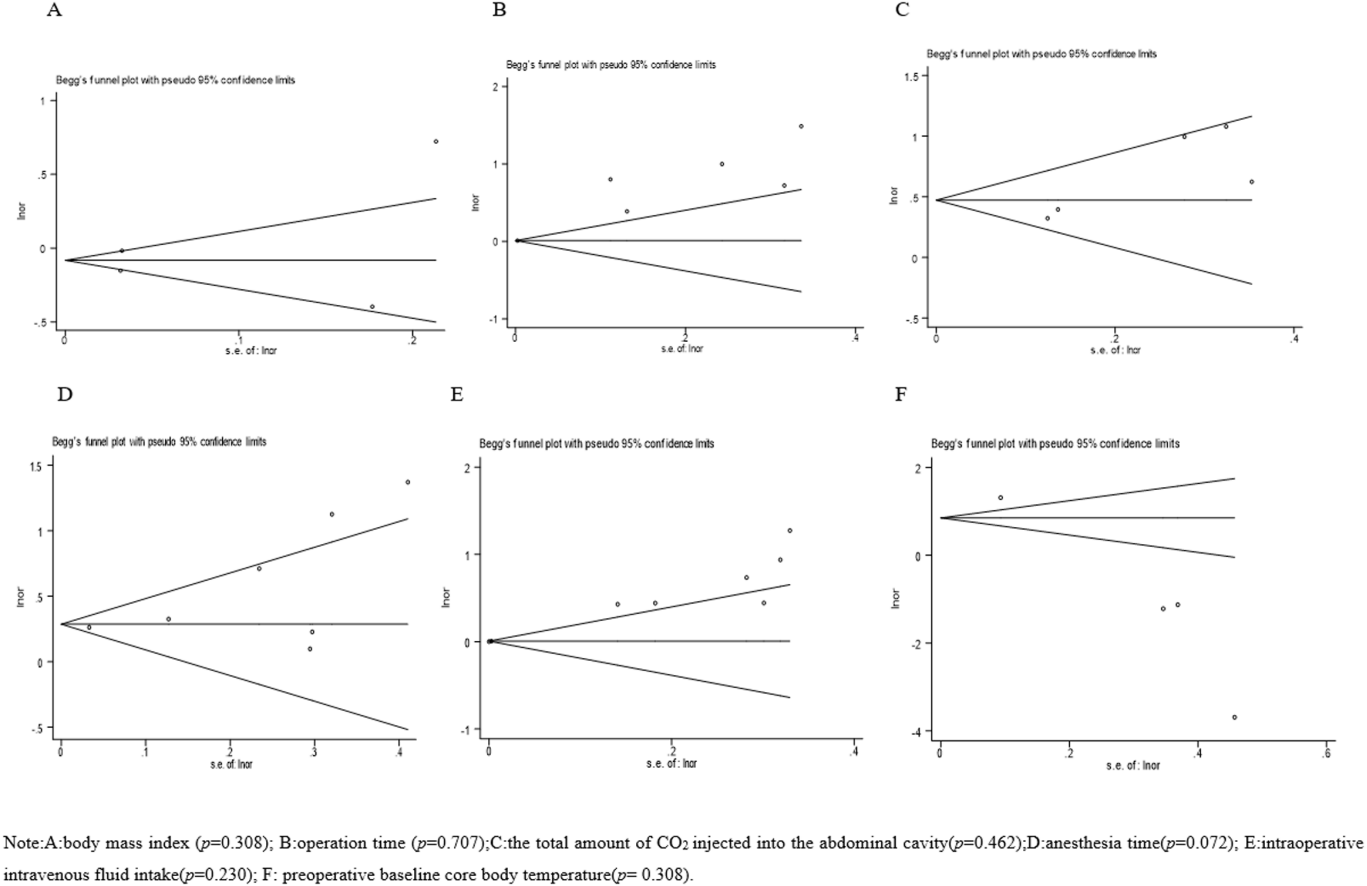
Publication bias funnel plot.

Only body mass index (BMI), operation time, and total CO2 injected into the abdominal cavity during the operation. Operation time, anesthesia time, intraoperative intravenous (IV)–fluid intake, and preoperative baseline core body temperature were analyzed for publication bias. (Fig 11).

## Discussion

After strict screening criteria were applied and both researchers independently evaluated the studies for quality, we included 11 studies in our meta-analysis. Of these studies, four were case–control and seven were cross-sectional. Sample sizes ranged from 62 to 690 cases, for a total of 3550 cases. We extracted 14 risk factors. In addition, guideline-recommended methods were used to measure hypothermia outcomes in surgical patients, and JBI tools for evaluating the quality of cohort and case–control studies were used to assess these studies. NOS scores were 6–7 and AHRQ scores 7–8, meaning that the quality of the included literature was good. Meta-analysis results showed that age, total amount of CO_2_ injected into the abdominal cavity during surgery, operation duration, anesthesia duration, amount of IV fluid administered for rehydration, and amount of blood lost during surgery were all high-risk factors for the occurrence of hypothermia.

### Risk factors

In this study, the results of two continuous-variable studies showed that age was an influential factor for hypothermia during laparoscopic surgery: the risk of hypothermia increased by 1.02× when the patient was ≥ 60 years of age. As a patient ages, the function and efficiency of body temperature regulation, vasoconstrictive response, total oxygen consumption, bodily heat production, and the ability to maintain core body temperature all decrease. Older people have less muscle mass and a decreased ability to generate heat through skeletal-muscle trembling. An elderly person’s ability to perceive cold and hot also decreases over time, decreasing their ability to maintain a stable body temperature [24]. The consensus among Chinese experts on the prevention of perioperative hypothermia also considers advanced age (> 60 years) a risk factor, which is consistent with the results of this study [25,26]. The older the surgical patient, the greater the incidence of hypothermia. It is therefore recommended that clinical staff begin to adopt protective measures for elderly surgical patients in the ward. Blankets and socks can be worn to help maintain baseline body temperature. In addition, reducing exposure of the body’s surface during surgical preparation and adjusting the ambient temperature of the operating room would be helpful. Preoperative pre-insulation measures were actively carried out, and heating equipment was actively applied during the operation to control the stability of core body temperature. Moreover, a series of comprehensive insulation measures such as hot-liquid infusion during surgery could reduce the occurrence of intraoperative hypothermia in patients and ensure their comfort during the procedure. Excessive intraoperative infusion can increase the risk of hypothermia, which might be related to heat conduction. Since the liquid transfused in the operating room is generally cooler than the body’s temperature, body heat can be transferred to it, thereby lowering core temperature. Moreover, with the increase of the cryogenic liquid transfused, especially when infusion volume > 1500 mL [27]. Some researchers have effectively reduced the incidence of chills and decreases in body temperature by preheating the infusion fluid [28]. Therefore, this method might enable medical staff to reduce the incidence of intraoperative hypothermia when intraoperative infusion volume cannot be reduced.

The risk of hypothermia increased 292× with total CO_2_ injection > 200 L, which might be related to the nature of CO_2_ drying (relative humidity is 0) and low temperature (20–21°C). The relationship between hypothermia and the amount of CO_2_ used in laparoscopy has attracted many scholars’ attention. To maintain positive abdominal pressure, a large amount of CO_2_ is used during laparoscopy. As the abdominal organs and blood vessels are extensively and continuously exposed to dryness and low-temperature CO_2_, the body warms and humidifies the gas via convection and conduction, thus lowering the body’s core temperature and causing intraoperative hypothermia [29]. Xu Cui *et al.* [30] and Oderda *et al.* [31] studied the effect on intraoperative body temperature of applying warmed and humidified CO_2_ to establish pneumoperitoneum. Their results showed that such a procedure could effectively reduce the degree of hypothermia and maintain a stable body temperature. These findings could provide guidance for the formulation of intraoperative interventional measures [32].

In the current study, we showed that duration of surgery was a major factor influencing the occurrence of hypothermia. The length of an operation determines the duration of anesthesia, and the degree to which body temperature can be controlled during anesthesia is limited. Therefore, the longer an operation, the longer the duration of anesthesia, and the greater the risk of hypothermia. Neuroanesthesia of major bodily organs or areas, such as the spine or epidural space, for >1 h leads to hypothermia. In general anesthesia, the patient’s thermoregulatory center is inhibited and peripheral-blood vessels are dilated, causing core temperature to drop rapidly within 1 h to 1.5°C. Later, because bodily heat production is less than heat dissipation, core temperature drops slowly and continuously to about 33°C before thermoregulation is activated. Then, heat production increases until the body reaches a plateau of thermoregulatory balance [33,34]. Due to these phenomena, our findings showed that operations lasting ≥ 120 min and ≥ 150 min increased the risk of hypothermia by 2.32× and 1.55 × , respectively. When conventional surgery must be prolonged due to the patient’s condition, the prolonged duration of anesthesia time increases the patient’s vulnerability to risk factors such as hypothermia [35,36]. Clinical nursing staff should therefore pay close attention to patients’ core body temperature during longer operations, implement nursing measures designed to prevent hypothermia, and actively warm the patient as needed. Such measures will greatly reduce the risk of hypothermia caused by prolonged operation time. The current study showed that intraoperative blood loss ≥ 150 mL was a risk factor for hypothermia. Moreover, laparoscopic surgery can damage platelet coagulatory function, reduce the activity of clotting factors, and increase blood loss [37,38]. Excessive blood loss, which is generally associated with longer operation time, affects arteriovenous blood circulation, thereby lowering core body temperature. Therefore, greater intraoperative blood loss can raise the risk of hypothermia. Furthermore, studies have shown that hypothermia can delay the initiation of the body’s coagulatory mechanism, thereby increasing intraoperative blood loss and the need for transfusion [39,40]. In this study, we also analyzed the effects of BMI, intraoperative irrigation volume, and preoperative baseline core body temperature on hypothermia. Although some individual studies showed that BMI and preoperative baseline core body temperature were risk factors for hypothermia, our meta-analysis showed no such statistical significance for these two factors, and therefore we could not conclude that they affected intraoperative hypothermia during laparoscopic surgery. However, the reasons for this should be considered. Notably, the number of included studies was too small and the heterogeneity too great; in addition, routine insulation measures taken during surgery might have influenced the results of the individual studies. We suggest that other scholars further this research to provide more-supportive evidence.

### Strengths and weaknesses

To the best of our knowledge, this study was the first to investigate the factors influencing intraoperative hypothermia in laparoscopy patients. The strength of our systematic review rested on the high quality of the included articles, providing it with a certain reference significance for future research. However, this study also had some limitations. First, most of the included studies were cohort and case–control studies, with only a few cross-sectional studies, rendering them susceptible to confounding factors that might have led to bias in the studies’ results. Second, the large differences in sample size and research tools might have led to publication bias. Third, we could find fewer than two studies on certain influencing factors, which made heterogeneity analysis impossible. We recommend that more high-quality original and prospective studies with greater sample sizes be conducted in the future to verify the stability of our results.

## Conclusions

This evidence-based meta-analysis explored different risk factors influencing the mechanism of hypothermia during laparoscopic surgery. The results showed that age, total amount of CO_2_ injected into the abdominal cavity during surgery, operation time, anesthesia time, amount of IV-fluid rehydration, and amount of blood loss during surgery had the highest correlations with the occurrence of hypothermia. Due to the small number of studies included and the great heterogeneity, we found insufficient evidence correlating BMI, intraoperative irrigation volume, and preoperative baseline core body temperature with the occurrence of hypothermia. More studies are needed to determine the effects of these factors.

The results of the current study can serve as a reference for high-quality, rigorous, and unified future studies to further confirm the factors that influence intraoperative hypothermia. Our findings will also provide practical value for medical staff in early diagnosis of and intervention into intraoperative hypothermia, so as to reduce its incidence as well as its adverse effects.

## Supporting information

S1 File PRISMA2020 checklist. (DOCX)

S2 File Characteristics of the included studies. (DOCX)

S3 File Quality assessment of research or results. (DOCX)

S4 File Literature search. (XLSX)
