# Supplementary material for: Risk factors for intraoperative hypothermia during laparoscopic surgery: A systematic review and meta-analysis
Source: PLoS One. 2025 Jul 17;20(7):e0328282. doi: 10.1371/journal.pone.0328282 (PMC12270140; doi:10.1371/journal.pone.0328282)
Supplement: S2 File — (DOCX) [file pone.0328282.s002.docx]

| Risk factors | Study | OR | 95% CI -LL | 95% CI -UL | β | Name of data extractors | date of data extraction |
| --- | --- | --- | --- | --- | --- | --- | --- |
| Age | Chen HL 2021 | 1.017 | 1.000 | 1.034 | 0.017 | Review Manager 5.4 a | July 1, 2024 |
|  | Fang M 2023 | 1.285 | 0.927 | 1.783 | 0.251 | Review Manager 5.4 | July 1, 2024 |
| Body mass index | Zhao D 2021 | 0.673 | 0.476 | 0.952 | -0.396 | Review Manager 5.4 | July 1, 2024 |
|  | Chen HL 2021 | 0.983 | 0.880 | 1.000 | -0.017 | Review Manager 5.4 | July 1, 2024 |
|  | Sung-Ae C 2022 | 0.859 | 0.807 | 0.914 | –0.152 | Review Manager 5.4 | July 1, 2024 |
|  | Shen CY 2024 | 2.061 | 1.413 | 3.263 | 0.115 | Review Manager 5.4 | July 1, 2024 |
| Total amount of CO_2_ injected into the abdominal cavity during the operation | Zhao Z 2018 | 1.867 | 1.059 | 4.223 | 0.693 | Review Manager 5.4 | July 1, 2024 |
|  | Ma GL 2020 | 2.944 | 1.560 | 5.559 | 1.080 | Review Manager 5.4 | July 1, 2024 |
|  | Zhao D 2021 | 1.382 | 1.083 | 1.764 | 0.324 | Review Manager 5.4 | July 1, 2024 |
|  | Liu L 2022 | 2.707 | 1.571 | 4.663 | 0.995 | Review Manager 5.4 | July 1, 2024 |
|  | Fang M 2023 | 1.486 | 1.138 | 1.940 | 0.396 | Review Manager 5.4 | July 1, 2024 |
| Operation time | Ma GL 2020 | 4.421 | 2.288 | 8.543 | 1.486 | Review Manager 5.4 | July 1, 2024 |
|  | Zhao D 2021 | 2.716 | 1.687 | 4.371 | 0.999 | Review Manager 5.4 | July 1, 2024 |
|  | Chen HY 2021 | 2.058 | 1.107 | 3.823 | 0.722 | Review Manager 5.4 | July 1, 2024 |
|  | Chen HL 2021 | 1.010 | 1.006 | 1.015 | 0.010 | Review Manager 5.4 | July 1, 2024 |
|  | Fang M 2023 | 1.474 | 1.140 | 1.906 | 0.388 | Review Manager 5.4 | July 1, 2024 |
|  | Shen CY 2024 | 2.228 | 1.925 | 2.981 | 0.189 | Review Manager 5.4 | July 1, 2024 |
| Duration of anesthesia | Zhao Z 2018 | 1.103 | 0.556 | 1.764 | 0.754 | Review Manager 5.4 | July 1, 2024 |
|  | Qi F 2019 | 1.300 | 1.172 | 1.334 | 0.159 | Review Manager 5.4 | July 1, 2024 |
|  | Pu Y 2019 | 1.256 | 1.009 | 3.234 | 0.009 | Review Manager 5.4 | July 1, 2024 |
|  | Ma GL 2020 | 3.083 | 1.645 | 5.779 | 1.126 | Review Manager 5.4 | July 1, 2024 |
|  | Zhao D 2021 | 2.036 | 1.286 | 3.223 | 0.711 | Review Manager 5.4 | July 1, 2024 |
|  | Liu L 2022 | 3.939 | 1.762 | 8.804 | 1.370 | Review Manager 5.4 | July 1, 2024 |
|  | Fang M 2023 | 1.385 | 1.080 | 1.777 | 0.326 | Review Manager 5.4 | July 1, 2024 |
| Intraoperative intravenous fluid intake | Zhao Z 2018 | 1.558 | 0.805 | 2.621 | 0.954 | Review Manager 5.4 | July 1, 2024 |
|  | Qi F 2019 | 1.006 | 1.001 | 1.011 | 0.006 | Review Manager 5.4 | July 1, 2024 |
|  | Ma GL 2020 | 3.574 | 1.873 | 6.820 | 1.274 | Review Manager 5.4 | July 1, 2024 |
|  | Zhao D 2021 | 1.556 | 1.089 | 2.224 | 0.442 | Review Manager 5.4 | July 1, 2024 |
|  | Chen HY 2021 | 2.554 | 1.366 | 4.773 | 0.938 | Review Manager 5.4 | July 1, 2024 |
|  | Sung-Ae C 2022 | 1.001 | 1.001 | 1.001 | 0.001 | Review Manager 5.4 | July 1, 2024 |
|  | Liu L 2022 | 2.084 | 1.199 | 3.624 | 0.734 | Review Manager 5.4 | July 1, 2024 |
|  | Fang M 2023 | 1.536 | 1.165 | 2.025 | 0.429 | Review Manager 5.4 | July 1, 2024 |
| Intraoperative irrigation volume | Chen HY 2021 | 1.746 | 1.079 | 2.826 | 0.558 | Review Manager 5.4 | July 1, 2024 |
|  | Chen HL 2021 | 1.001 | 1.000 | 1.001 | 0.001 | Review Manager 5.4 | July 1, 2024 |
| Intraoperative blood loss | Ma GL 2020 | 5.768 | 3.069 | 10.840 | 1.752 | Review Manager 5.4 | July 1, 2024 |
|  | Liu L 2022 | 2.053 | 1.187 | 3.550 | 0.719 | Review Manager 5.4 | July 1, 2024 |
|  | Fang M 2023 | 1.554 | 1.145 | 2.110 | 0.441 | Review Manager 5.4 | July 1, 2024 |
| Preoperative baseline core body temperature | Pu Y 2019 | 0.296 | 0.253 | 0.982 | -1.216 | Review Manager 5.4 | July 1, 2024 |
|  | Chen HL 2021 | 0.025 | 0.010 | 0.060 | -3.689 | Review Manager 5.4 | July 1, 2024 |
|  | Sung-Ae C 2022 | 0.324 | 0.157 | 0.666 | -1.128 | Review Manager 5.4 | July 1, 2024 |
|  | Shen CY 2024 | 3.715 | 3.011 | 4.335 | 0.107 | Review Manager 5.4 | July 1, 2024 |
