# Supplementary material for: Risk factors for intraoperative hypothermia during laparoscopic surgery: A systematic review and meta-analysis
Source: PLoS One. 2025 Jul 17;20(7):e0328282. doi: 10.1371/journal.pone.0328282 (PMC12270140; doi:10.1371/journal.pone.0328282)
Supplement: S3 File — (DOCX) [file pone.0328282.s003.docx]

| **Author,year** | ① | ② | ③ | ④ | ⑤ | ⑥ | ⑦ | ­⑧ | **Scores** |
| --- | --- | --- | --- | --- | --- | --- | --- | --- | --- |
| Qi F 2019[14] | 1 | 1 | 1 | 1 | 0 | 1 | 1 | 0 | 6 |
| Pu Y 2019[15] | 1 | 1 | 1 | 1 | 1 | 1 | 1 | 0 | 7 |
| Ma GL 2020[16] | 1 | 1 | 1 | 1 | 1 | 1 | 1 | 1 | 7 |
| Zhao D 2021[17] | 1 | 1 | 1 | 1 | 0 | 1 | 1 | 1 | 7 |
| Sung-Ae C 2022[20] | 1 | 1 | 1 | 1 | 1 | 1 | 1 | 0 | 7 |
| Liu L 2022[21] | 1 | 1 | 1 | 1 | 1 | 1 | 1 | 0 | 7 |
| Fang M 2023[22] | 1 | 1 | 1 | 1 | 0 | 1 | 1 | 0 | 6 |

**Methodological quality evaluation of observational studies**

Note：1 indicates yes; 0 indicates no or unclear. ①Representation of the exposed cohort；②Representation of the non-exposed cohort；③Methods for determining exposure factors；④No outcome indicator had occurred before study initiation；⑤Comparability of exposed and non-exposed queues；⑥Evaluation of outcome indicators；⑦For the subjects studied, whether the follow-up time is long enough；⑧Adequacy of follow-up

| **Author,year** | ① | ② | ③ | ④ | ⑤ | ⑥ | ⑦ | ­⑧ | ⑨ | ⑩ | ⑪ | **Scores** |
| --- | --- | --- | --- | --- | --- | --- | --- | --- | --- | --- | --- | --- |
| Zhao Z 2018[13] | 1 | 1 | 1 | 1 | 0 | 1 | 1 | 0 | 1 | 0 | 0 | 7 |
| Chen HY 2021[18] | 1 | 1 | 1 | 1 | 0 | 1 | 1 | 1 | 1 | 0 | 0 | 8 |
| Chen HL 2021[19] | 1 | 1 | 1 | 1 | 1 | 0 | 1 | 1 | 1 | 0 | 0 | 8 |
| Shen CY 2024[23] | 1 | 1 | 1 | 1 | 1 | 0 | 1 | 1 | 0 | 0 | 0 | 7 |

**Methodological quality evaluation of cross-sectional studies**

Note：1 indicates yes; 0 indicates no or unclear. ①Define the source of information; ②List inclusion and exclusion criteria for exposed and unexposed subjects (cases and controls) or refer to previous publications; ③Indicate time period used for identifying patients; ④Indicate whether or not subjects were consecutive if not population-based; ⑤ Indicate if evaluators of subjective components of study were masked to other aspects of the participants; ⑥Describe any assessments undertaken for quality assurance purposes (e.g.,test/retest of primary outcome measurements); ⑦Explain any patient exclusions from analysis; ⑧Describe how confounding was assessed and/or controlled; ⑨ If applicable, explain how missing data were handled in the analysis; ⑩ Summarize patient response rates and completeness of data collection; ⑪ Clarify what follow-up, if any, was expected and the percentage of patients for which incomplete data or follow-up was obtained
